# Effects of Early Life Stress on Synaptic Plasticity in the Developing Hippocampus of Male and Female Rats

**DOI:** 10.1371/journal.pone.0164551

**Published:** 2016-10-10

**Authors:** Nienke A. V. Derks, Harm J. Krugers, Casper C. Hoogenraad, Marian Joëls, R. Angela Sarabdjitsingh

**Affiliations:** 1 Department of Translational Neuroscience, Brain Center Rudolf Magnus, University Medical Center Utrecht, Utrecht, The Netherlands; 2 Swammerdam Institute for Life Sciences-Center for Neuroscience, University of Amsterdam, Amsterdam, The Netherlands; 3 Cell Biology, Department of Biology, Faculty of Science, Utrecht University, Utrecht, The Netherlands; 4 University Medical Center Groningen, Groningen, The Netherlands; Technion Israel Institute of Technology, ISRAEL

## Abstract

**Introduction:**

Early life stress (ELS) increases the risk for developing psychopathology in adulthood. *When* these effects occur is largely unknown. We here studied at which time during development ELS affects hippocampal synaptic plasticity, from early life to adulthood, in a rodent ELS model. Moreover, we investigated whether the sensitivity of synaptic plasticity to the stress-hormone corticosterone is altered by exposure to ELS.

**Materials & Methods:**

Male and female Wistar rats were exposed to maternal deprivation (MD) for 24h on postnatal day (P)3 or left undisturbed with their mother (control). On P8-9, 22–24 and P85-95, plasma corticosterone (CORT) levels, body weight, and thymus and adrenal weights were determined to validate the neuroendocrine effects of MD. Field potentials in the CA1 hippocampus were recorded *in vitro* before and after high frequency stimulation. Brain slices were incubated for 20 min with 100nM CORT or vehicle 1-4h prior to high frequency stimulation, to mimic high-stress conditions *in vitro*.

**Results & Discussion:**

Body weight was decreased by MD only at P4 (p = 0.02). There were minimal effects on P8-9, 22–24 or 85–95 thymus and adrenal weight and basal CORT levels. Glutamate transmission underwent strong developmental changes: half-maximal signal size strongly increased (p<0.0001) while the required half-maximal stimulation intensity concomitantly decreased with age (p = 0.04). Synaptic plasticity developed from long-term depression at P8-9 to increasing levels of long-term potentiation at later ages (p = 0.0001). MD caused a significant increase in long-term potentiation of P22-24 males (p = 0.03) and P85-95 females (p = 0.04). Bayesian modeling strongly supported the age-dependent development, with some evidence for accelerated maturation after MD in males (Bayes factor 1.23). CORT suppressed LTP in adult males; synaptic plasticity at other ages and in females remained unaffected. Thus, MD affects the development of synaptic plasticity in the CA1 hippocampus in a sex-dependent manner, with some support for the notion that maturation is accelerated in MD males.

## Introduction

Early life adversities such as physical or emotional abuse, neglect or loss of a parent increase the risk of developing psychopathology later in life[1–4] and are highly prevalent[5,6]. Altered programming of the stress system[7,8] and brain development[9] by early stress are thought to be major contributors to the risk for psychopathology[10]. Yet, the exact mechanisms by which these alterations after childhood adversities contribute to the development of psychopathology are highly complex and hard to study in humans.

In order to study the mechanisms by which early life stress (ELS) alters brain functioning, animal models are widely used. One of the models used is maternal deprivation (MD) at postnatal day (P)3, when the dam is removed from the pups for 24 hours[11]. Prolonged deprivation of pups from their mother activates the HPA axis and thereby the release of corticosterone (CORT) during the stress hyporesponsive period[12]. From previous studies, mostly in adult organisms, it has become evident that MD affects neuronal morphology and synaptic transmission in multiple brain areas[13–15]. Since the hippocampus is still in development during the first postnatal weeks in rodents[16] and has a high expression of both mineralocorticoid as well as glucocorticoid receptors (MR and GR respectively), this brain area is likely to be highly susceptible to early external influences.

Many studies have examined the effects of MD on behavioral or cellular function in the adult hippocampus. Yet, the appearance of effects by MD is highly age-dependent: different effects have been found when testing the animals in behavioral/memory tasks or neurogenesis as young adults, adults or in senescence[17–19]. In order to get a better impression of *when* MD affects brain functioning, it is of critical importance to understand how parameters of interest are affected during different stages of development.

Besides age-dependent effects of MD, sex differences have also been described, in line with human data[20,21]. In rats, MD on P3 impaired spatial memory in adult males but not in females, and males showed a stronger freezing response in fear conditioning. Also, dentate morphology and synaptic plasticity were differently affected by MD in adult males and females.

A final consideration is the plasticity of hippocampal cells in neutral as well as in high-stress conditions. According to the match-mismatch hypothesis, an individual should perform optimally when the adult context matches the early life environment[22]. In line with this hypothesis, we expect MD animals to show efficient synaptic plasticity when tested under high-stress conditions -mimicked *in vitro* by CORT incubation- as opposed to conditions characterized by low CORT levels. This interplay between early life environment and CORT level on hippocampal plasticity has been demonstrated in earlier studies investigating MD[13] or other models of early life adversity[23,24].

In the current study we investigated *when* MD affects synaptic plasticity of CA1 hippocampal pyramidal neurons throughout development, in both male and female rats. Specifically, we studied 1) the effect of MD on baseline field excitatory postsynaptic potential (fEPSP) properties and synaptic plasticity at different stages of development, 2) whether these effects are sex-dependent, and 3) how hippocampal fEPSP properties respond to high-stress CORT conditions, mimicked *in vitro* by incubation with 100 nM CORT[13]. We focused on three developmental stages relevant for stress effects on hippocampal physiology: the start of the second postnatal week, when a critical period for epigenetic changes has just closed[25]; the juvenile period, which is shown to be a critical time window for (re)programming of the HPA-axis[26] and during which the largest changes in neurogenesis have been found (at least in females)[19]; and early adulthood, when MD exposed rats show altered learning and memory performance[13,14].

## Materials and Methods

### Animals and Breeding Procedure

All animal procedures described in this article were approved by the local committee for Animal Health, Ethics and Research of Utrecht University (Utrecht, The Netherlands). Nulliparous female and male Wistar rats were ordered from Charles River Laboratories (Arbresle, France) at 10 weeks of age. Animals were kept under standard housing conditions (12/12h light/dark cycle, lights on at 7:00 A.M., temperature 20–22°C, humidity 55±15%, *ad libitum* access to food and water) and were allowed to habituate to the animal facility for at least 1 week prior to breeding.

Two females were housed together with one male for 10 days, after which the male was removed from the cage. Males were used again for multiple breedings. In the third gestational week, the females were individually housed and provided with nesting material. Females were daily checked for birth before 9:00 A.M. If a litter was found, the previous day was assigned to be P0. At P3, litters were culled to a maximum of 10 pups, keeping the male:female ratio as equal as possible. Litters smaller than 6 pups were not used for experiments.

### Maternal Deprivation Procedure

Litters were randomly assigned to either the MD or control group. Between 10:00 and 11:00 A.M. on P3, dams of MD litters were placed in a new cage without nesting material. The pups remained together in their home cage and were moved to an adjacent room. During the 24h deprivation period, litters were kept in the dark at a constant temperature of 34°C. On the morning of P4, the litter was returned to the breeding room and reunited with the dam. Pups were weaned at P21 and housed together with same-sex littermates (2–4 animals per cage). The last remaining animal of each cage was not used for experiments to prevent effects of stress by solitary housing.

### Tissue Preparation

**Fig 1:**
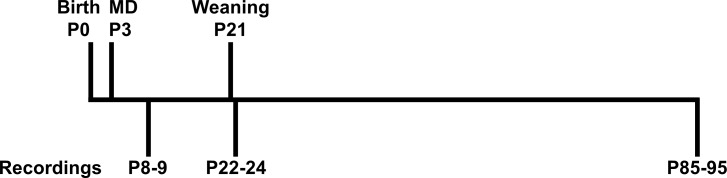
Experimental design. Half of the litters were maternally deprived for 24h at postnatal day (P)3. At P8-9, 22–24 and 85–95, animals were used for field potential recordings in the CA1 hippocampus.

At the day of the experiment, young (P8-9), juvenile (P22-24) and (young) adult (P85-95) male and female rats were weighed and decapitated early in the morning between 8:30 and 10:00 A.M., when endogenously circulating CORT levels are still low (see Fig 1 for experimental setup). Trunk blood was collected in heparin-coated tubes and the thymus and adrenals were dissected to assess the effects of MD on these stress-sensitive parameters. Trunk blood was centrifuged (10,000 rpm, 10 min at room temperature) and plasma was stored at -20°C until CORT levels were determined *in duplo* with a commercially available radioactive immunoassay (MP Biomedicals, United States of America). Vaginal smears were taken from adult female rats after decapitation, after which Giemsa staining was performed to assess the stage of the estrus cycle.

Brains were rapidly dissected and placed in ice-cold modified artificial cerebrospinal fluid (aCSF) containing (in mM): 120 choline chloride, 3.5 KCl, 6 MgSO_4_, 1.25 NaH_2_PO_4_, 0.5 CaCl_2_, 10 glucose and 25 NaHCO_3_, continuously gassed with a mixture of 95% O_2_ and 5% CO_2_. Using a vibratome (LEICA VT 1000S, Germany), 500 μm (P8-9 brains) or 350 μm (other ages) coronal slices were made containing the dorsal hippocampus. Slices were transferred to aCSF containing 120 NaCl, 3.5 KCl, 1.3 MgSO_4_, 1.25 NaH_2_PO_4_, 2.5 CaCl_2_, 10 glucose and 25 NaHCO_3_ for 20 min at 32°C to recover from the slicing procedure. Subsequently, slices were stored in a holding chamber at room temperature for at least 1h. Slices were incubated for 20 min at 32°C in either aCSF containing 100nM CORT dissolved in 0.01% ethanol or vehicle (VEH, 0.01% ethanol). All chemicals were from Sigma-Aldrich (Germany).

### Field Potential Recordings

After incubation of slices with CORT or VEH, one brain slice at a time was moved to a recording chamber with constant perfusion of aCSF (32°C, flow rate 1.2–1.5 ml/min (but 3 ml/min for P8-9 slices)). fEPSPs were recorded in the Schaffer collateral-CA1 pathway as described previously[27]. In short, a bipolar stimulation electrode (CBBRC75, FHC, USA) was placed on the Schaffer collaterals and a glass recording pipette filled with aCSF (resistance 3–6 MΩ) positioned in the CA1 stratum radiatum. When a stable signal was found, the (half)maximum slope or amplitude and corresponding stimulation intensities were determined by making an input-output curve (0.067 Hz, stimulus duration 0.15 ms). For dendritic fEPSP recordings, measurement of the slope is the preferred approach, mostly because amplitude measurements can be confounded by a reflection of the somatic compound action potential. However, amplitude recordings are generally more stable and easier to determine, and therefore preferable when there is no confounding action potential. This appeared to be the case for recordings from P8-9 animals: signals were extremely small (see Results section), with no confounding action potentials. Thus, amplitude measurements for this group were more reliable than determination of the slope. For the two other age-groups we determined the slope of the fEPSP signals.

Baseline synaptic transmission was recorded with the half-maximum stimulation intensity for 10 min (0.033 Hz, duration 0.10 ms), after which high-frequency stimulation (HFS, 10 Hz, 90s, duration 0.15 ms per stimulus) was applied. HFS was always applied between 1 and 4 hours after the start of a 20 min VEH or CORT incubation in order to investigate the slow, genomic effects of CORT. Post-HFS transmission was recorded for 60 min with the same settings as the baseline recording. At the end of the post-HFS recording, a second input-output was made to determine changes in signal properties. Data were analyzed off-line using Signal 2.16 (Cambridge 159 Electronic Design, UK).

### Statistical Analysis

Statistical analyses were performed with Graphpad Prism 6.07 and IBM SPSS Statistics 23.0. Group values are expressed as mean±SEM. Basal plasma CORT levels and body weight were compared within each age and sex category between MD and control groups by two-tailed independent samples t-tests. Thymus weight and adrenal weight were both expressed as g/100g body weight and tested in the same way as plasma CORT and body weight. In addition, a linear regression analysis was performed to correlate basal plasma CORT levels in trunk blood with synaptic plasticity.

Input-output curves were fitted offline with Boltzmann equations in ClampFit 8.2 and first tested for the effect of age in males and females separately (one-way ANOVA), followed by a secondary analysis within each age and sex category with a two-way ANOVA (treatment x incubation). In addition, changes in the input-output parameters (i.e. half-maximal stimulus intensity, maximal response and slope of the Boltzmann curve) were tested by comparing the input-output curves before and after applying HFS with a paired-samples t-test. Baseline fEPSP slopes over 10 min (prior to HFS) were averaged and set to 100%. Post-HFS recordings were expressed as a percentage of the baseline recording to determine the change in synaptic strength. With a paired samples t-test, we determined whether the HFS induced a significant change in fEPSP slope compared to baseline. Post-HFS slopes (% of baseline) were compared for the effects of treatment and incubation with two-way ANOVAs for each age-group and sex. In an exploratory analysis we also examined the effect of VEH versus CORT incubation *within* animals with a paired samples t-test, to allow comparison with earlier studies with this model. In all cases, P-values <0.05 were considered to be significantly different.

To evaluate how synaptic plasticity develops with age and how MD affects this development, we used a Bayesian approach, since the application of generalized estimating equations assumes linearity, which clearly is not the case in the MD group. With Bayesian statistics, one is able to test a specific informative hypothesis about how groups are related against the unconstrained alternative hypothesis ‘Any ordering of the group means is equally likely’[28]. In this way, one gains in power by directly testing the probability of the *a priori* formulated hypothesis of interest. Instead of rejecting a null hypothesis, Bayesian statistics indicate how much the data support one hypothesis over another. This can be compared with either an unconstraint alternative hypothesis or with multiple other formulated hypotheses.

Based on previous literature, we formulated specific hypotheses about the expected developmental effect on synaptic plasticity. We expected to observe long-term depression (LTD) after HFS in P8-9 slices, since the signaling circuitry at this age is still in a developmental state that favors LTD induction[29,30]. In contrast, long-term potentiation (LTP) was observed earlier in adult mice in studies using the same stimulation protocol as described here[27]. When comparing juveniles with adults, short-term plasticity in the Schaffer-collateral pathway showed enhanced facilitation with age[31]. Therefore, we expected LTP to follow the same pattern. To predict the effects of MD on development, we based our hypothesis on data from Bath et al.[32]. In mice subjected to limited nesting and bedding material early in life, developmental hallmarks such as a decline in markers for cell proliferation and expression, NMDA receptor subunit expression and the developmental suppression of contextual fear were all accelerated by early life stress. Hypothesizing that LTP follows the same pattern after MD in rats, we expected a plateau effect in the magnitude of induced LTP in adults.

Four hypotheses on developmental effects of synaptic plasticity and interference of MD were tested: (1) Increased synaptic strength after HFS with age in both controls and MD, (2) increase from P8-9 to P22-24, plateau effect in P22-24 to P85-95 in both controls and MD, (3) increase in control, plateau in MD and (4) plateau in control, increase in MD. An unconstraint hypothesis, assuming no particular ordering of the group means, was added as a control model. Bayesian analyses were performed with BIEMS software (Bayesian Inequality and Equality constrained Model Selection)[33]. Output of Bayesian analyses include a Bayes factor (indicating a relative measure of support for a hypothesis) and a posterior model probability (PMP, stating the probability of a hypothesis within the set of tested hypotheses, adding up to 1 (i.e. 100%) in total).

## Results

### Effects of MD on Neuroendocrine Parameters

To validate the effectiveness of the MD model, we determined both immediate and long-term effects of MD on several stress-sensitive parameters such as basal plasma CORT, body weight, thymus weight and adrenal weight. As expected, there was an immediate decrease in body weight by MD in both male and female pups at P4 (Table 1, males p = 0.02, females p = 0.02). This effect was transient as it was not observed from P8-9 onwards (males p = 0.14, females p = 0.32). Basal CORT levels were measured in plasma collected in the morning, when CORT levels are usually low. MD did not affect basal CORT levels at any age, although there was a trend towards increased basal CORT levels in adult MD females (p = 0.07). Interestingly, plasma CORT levels were relatively high at P22-24. Thymus weight and adrenal weight were assessed on P8-9, 22–24 and 85–95. Adrenal weight was comparable for MD and control animals, at all ages. Thymus weight was decreased in P8-9 MD males compared to control males of the same age (p = 0.03), but not at other ages or in females. In sum, MD only transiently affected (some of) the stress-sensitive parameters.

**Table 1:** Decrease of body, thymus and adrenal weight after MD.

Age	Sex	Group	Body weight (g)	n	Thymus weight (mg/100g BW)	n	Adrenal weight (mg/100g BW)	n	ng CORT/ml plasma	n
P4	M	Control	9.2 ± 0.4	4						
		MD	7.9 ± 0.2 *	10						
	F	Control	8.6 ± 0.2	8						
		MD	7.9 ± 0.2 *	8						
P8-9	M	Control	15.0 ± 1.0	5	345.6 ± 10.5	5	22.1 ± 1.3	5	4.0 ± 0.8	6
		MD	13.5 ± 0.4	7	284.7 ± 18.3 *	7	21.0 ± 1.1	7	5.9 ± 1.7	9
	F	Control	15.8 ± 1.1	6	367.7 ± 19.7	6	21.9 ± 0.7	6	4.9 ± 0.8	9
		MD	14.4 ± 0.8	6	321.1 ± 32.7	6	23.8 ± 1.3	6	5.4 ± 1.0	8
P22-24	M	Control	47.9 ± 1.4	15	388.7 ± 13.1	15	32.0 ± 1.0	15	76.9 ± 15.0	14
		MD	45.4 ± 0.8	13	408.4 ± 14.4	13	34.1 ± 1.2	13	128.5 ± 31.5	13
	F	Control	45.8 ± 1.9	12	435.9 ± 12.2	8	33.8 ± 2.1	11	102.8 ± 21.9	11
		MD	44.0 ± 1.2	11	453.8 ± 16.6	11	37.1 ± 1.0	11	102.9 ± 30.7	13
P85-95	M	Control	333.0 ± 6.3	15	133.2 ± 4.1	13	13.6 ± 0.2	13	13.0 ± 6.7	12
		MD	341.0 ± 3.5	13	130.7 ± 3.7	13	13.6 ± 0.2	13	6.4 ± 1.9	16
	F	Control	215.9 ± 2.9	18	195.1 ± 6.4	16	24.1 ± 0.3	16	26.9 ± 7.5	17
		MD	209.9 ± 3.9	9	196.4 ± 9.4	8	24.6 ± 0.7	7	53.6 ± 13.5	10

Body weight was decreased directly after MD in both males (p = 0.02) and females (p = 0.02). Organ weights, body weight and basal CORT levels were not assessed at P4. Besides the decrease in thymus weight on P8-9 MD males compared to controls, animals did not show long-lasting effects of MD on stress-sensitive parameters. Mean ± SEM

*p<0.05.

### Developmental and MD Effects on Basal Field Potential Properties

We first assessed the effect of age on baseline properties of CA1 fEPSPs, based on input-output curves fitted with a Boltzmann equation. Data from P8-9 males and females were pooled after we verified that at this age, there was no effect of sex on basal properties (half-maximum signal size males 0.20 ± 0.04 μV vs females 0.21 ± 0.03 μV, p = 0.83, stimulation intensity 2.5 ± 0.1 V vs 2.7 ± 0.1 V, p = 0.29) or synaptic plasticity (mean last 10 min post-HFS recording 88.6 ± 4.7% vs 96.9 ± 7.3%, p = 0.48, see below).

**Fig 2:**
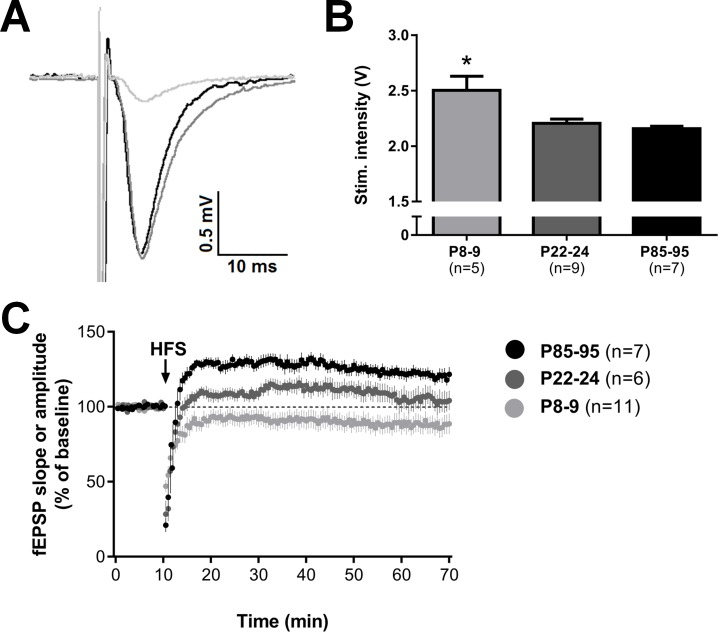
Basal field potential properties and LTP strongly increase during development. With age, the baseline halfmaximum signal amplitude strongly increased (A, p<0.0001), the required stimulation intensity decreased (B, p = 0.04) and the response to high-frequency stimulation developed from long-term depression at P8-9 to increasing levels of long-term potentiation with age (C, mean 60 min post-HFS, p = 0.0001). Data are on based on data obtained in the control male group, except for P8-9 where data from males and females were pooled.

Both baseline fEPSP amplitude and the required stimulation intensity showed clear developmental changes. In control VEH males, half-maximum signal size strongly increased with age (Fig 2, p<0.0001). Post-hoc analysis indicated that this increase takes place from P8-9 to P22-24. Concomitantly, half-maximum stimulation intensity decreased (p = 0.04). Similar effects of age on half-maximum signal size and stimulation intensity were seen for all other groups (Table 2).

**Table 2:** Boltzmann-fitted basal properties of recorded field potentials.

Age	Sex	Group	n	Halfmax amplitude (μV) or slope (mV/ms) PRE	Halfmax amplitude (μV) or slope (mV/ms) POST	Halfmax stimulation intensity (V) PRE	Halfmax stimulation intensity (V) POST	Slope IO curve PRE	Slope IO curve POST
P8-9	M+F	ctrl VEH	9	0.25 ± 0.03 μV	0.20 ± 0.02 μV	2.22 ± 0.04	2.30 ± 0.03	0.178 ± 0.029	0.200 ± 0.037
		ctrl CORT	8	0.24 ± 0.03 μV	0.23 ± 0.03 μV	2.23 ± 0.04	2.30 ± 0.03	0.174 ± 0.016	0.185 ± 0.021
		MD VEH	11	0.28 ± 0.04 μV	0.27 ± 0.04 μV	2.20 ± 0.04	2.33 ± 0.05	0.131 ± 0.015	0.185 ± 0.039
		MD CORT	11	0.30 ± 0.06 μV	0.27 ± 0.05 μV	2.24 ± 0.05	2.36 ± 0.05	0.144 ± 0.019	0.141 ± 0.014
P22-24	M	ctrl VEH	9	0.64 ± 0.07	0.62 ± 0.07	2.14 ± 0.03	2.15 ± 0.03	0.100 ± 0.013	0.100 ± 0.012
		ctrl CORT	10	0.64 ± 0.07	0.59 ± 0.05	2.10 ± 0.02	2.13 ± 0.01	0.100 ± 0.009	0.100 ± 0.006
		MD VEH	8	0.68 ± 0.09	0.70 ± 0.11	2.09 ± 0.02	2.12 ± 0.03	0.116 ± 0.012	0.108 ± 0.014
		MD CORT	6	0.54 ± 0.08	0.61 ± 0.07	2.13 ± 0.04	2.14 ± 0.04	0.123 ± 0.019	0.130 ± 0.029
P22-24	F	ctrl VEH	8	0.54 ± 0.06	0.56 ± 0.06	2.10 ± 0.02	2.13 ± 0.02	0.094 ± 0.009	0.087 ± 0.010
		ctrl CORT	10	0.52 ± 0.08	0.54 ± 0.09	2.10 ± 0.02	2.12 ± 0.02	0.055 ± 0.040	0.071 ± 0.027
		MD VEH	8	0.64 ± 0.06	0.71 ± 0.08	2.11 ± 0.02	2.13 ± 0.03	0.109 ± 0.016	0.098 ± 0.013
		MD CORT	5	0.53 ± 0.09	0.53 ± 0.04	2.11 ± 0.03	2.10 ± 0.03	0.110 ± 0.013	0.089 ± 0.023
P85-95	M	ctrl VEH	7	0.65 ± 0.06	0.71 ± 0.04	2.07 ± 0.02	2.09 ± 0.02	0.100 ± 0.021	0.086 ± 0.017
		ctrl CORT	7	0.67 ± 0.06	0.61 ± 0.05	2.08 ± 0.03	2.11 ± 0.01	0.126 ± 0.024	0.082 ± 0.010 *
		MD VEH	7	0.65 ± 0.03	0.65 ± 0.09	2.11 ± 0.02	2.09 ± 0.03	0.115 ± 0.022	0.088 ± 0.014
		MD CORT	8	0.68 ± 0.04	0.63 ± 0.06	2.02 ± 0.01 *	2.01 ± 0.01	0.061 ± 0.010	0.040 ± 0.007 *
P85-95	F	ctrl VEH	14	0.62 ± 0.04	0.66 ± 0.05	2.06 ± 0.02	2.09 ± 0.02	0.076 ± 0.020	0.078 ± 0.017
		ctrl CORT	10	0.73 ± 0.06	0.77 ± 0.07	2.10 ± 0.02	2.10 ± 0.02	0.103 ± 0.010	0.092 ± 0.011
		MD VEH	11	0.55 ± 0.06	0.65 ± 0.06	2.07 ± 0.02	2.06 ± 0.02	0.093 ± 0.007	0.077 ± 0.010
		MD CORT	6	0.64 ± 0.06	0.68 ± 0.05	2.11 ± 0.02	2.11 ± 0.02	0.118 ± 0.020	0.095 ± 0.015

Input-output curve data was fitted with the Boltzmann equation and averaged per group. Values represent mean ± SEM. IO curve slopes are shown as normalized values between 0 and 1. In P85-95 males, combined MD and CORT reduced the halfmaximum stimulation intensity pre-HFS (interaction effect, p = 0.03). CORT decreased the slope of the input-output curve post-HFS in this group (main effect, p = 0.04).

* indicates a significant effect (p<0.05).

Next we examined whether MD and/or CORT affect baseline transmission of CA1 fEPSPs. In view of the age-dependency of baseline properties, we analyzed the 4 experimental groups for each age separately, both in males and females (except for P8-9 where data were pooled). In nearly all age-groups, we did not observe an effect of either MD, CORT or an interaction effect on maximum signal size, half-maximum stimulation intensity or IO-curve slope (Table 2). However, in P85-95 males, the stimulation intensity required to evoke a half-maximum signal was lower in the MD CORT condition (interaction effect, p = 0.03) and CORT decreased the slope of the post-HFS input-output curve (main effect, p = 0.04). Overall, though, neither MD nor CORT had strong effects on baseline fEPSP characteristics.

### Developmental and MD Effects on Synaptic Plasticity

We observed a strong developmental effect on the response to HFS in control VEH males. In P8-9 animals, HFS overall induced a clear trend towards LTD (mean amplitude during last 10 minutes post-HFS recording vs baseline, p = 0.06). Instead, increasing levels of LTP were seen in P22-24 to P85-95 (mean slope during last 10 min post-HFS recording vs baseline, p = 0.30 and p<0.01 respectively). This age-dependent development in response to HFS was statistically significant (one-way ANOVA, p<0.002). In control VEH females, the trend towards LTD was followed by a significant but short-lasting potentiation at P22-24 (middle 20 min post-HFS recording vs baseline p = 0.0004, last 10 min vs baseline p = 0.20). In accordance with the male data, stable LTP was induced in adult females (p = 0.0001) and synaptic strength after HFS increased with age (one-way ANOVA, p = 0.005). Before examining the condition x treatment interactions in the various age groups, we tested the effect of CORT treatment alone in control males and females, to allow comparison wither earlier studies on the effectiveness of the drug. CORT was found to suppress LTP induction in P85-95 males, as reported before[34–36] (mean last 10 min post-HFS recording, p<0.05). CORT did not alter levels of synaptic plasticity in any of the other groups, although there was a trend (p = 0.07) in P85-95 females towards a suppression of LTP induction by CORT.

**Fig 3:**
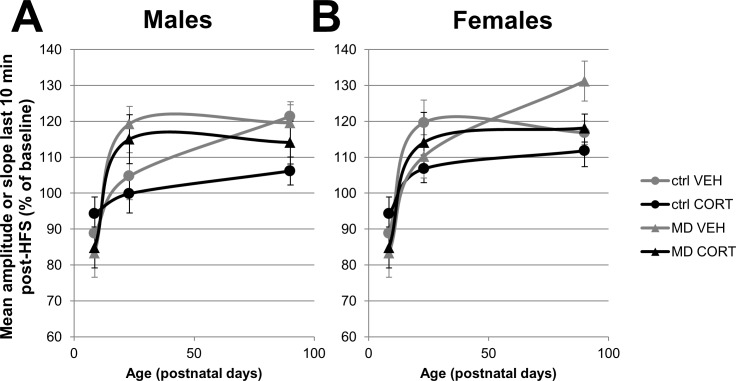
Influences of MD and CORT on the development of synaptic plasticity in the CA1 hippocampus in males and females. The response to high-frequency stimulation (HFS) is shown as the mean signal amplitude (P8-9) or slope (other ages) during the last 10 minutes of the 60 min post-HFS recording (± SEM). Male and female data on P8-9 was pooled after being tested for sex effects on fEPSP baseline characteristics and synaptic plasticity. (A) In males, control animals showed an increase in response to HFS which continued up into adulthood, while MD animals reached adult levels of long-term potentiation already at P22-24. (B) In females, controls showed a plateau effect in the development of synaptic strength. MD did not accelerate maturation in this case, but instead showed an even further increased magnitude of LTP induction.

We next examined the potential condition x treatment interactions for the three age groups, in males and females separately (except for P8-9 where data were pooled; Fig 3). Two-way ANOVA did not yield significant interaction effects nor main effects of CORT treatment, yet indicated a main (facilitatory) effect of MD in P22-24 males (p = 0.03) and P85-95 females (p = 0.04). With regard to the P85-95 females, all stages of the estrus cycle were pooled after verifying that there was no effect of estrus cycle on LTP-induction (one-way ANOVA, p = 0.88). All in all, both age and early life experience affected synaptic plasticity in the CA1 hippocampus, in a sex-specific manner.

In view of the significant group effect of CORT in P85-95 male rats, we performed a linear regression analysis with plasma CORT level as fixed variable and %LTP during the last 10 min of post-HFS recording as the dependent variable. Before fitting a line, outliers were automatically detected using the ROUT method[37]; three outliers were removed from the dataset (of which one in the P85-95 males). We tested whether the slope of the fitted line was significantly different from zero (i.e. whether there was a significant correlation between the two factors). Although we only had a small sample (n = 4), we observed a negative correlation between %LTP and plasma CORT (p = 0.004).

We next tested multiple hypotheses about the development of synaptic plasticity and the interference by MD with Bayesian statistics (see Methods section for details). In short, these hypotheses included: (1) Increased synaptic strength after HFS with age in both controls and MD, (2) increase from P8-9 to P22-24, plateau-effect in P22-24 to P85-95 in both controls and MD, (3) increase in control, plateau in MD, (4) plateau in control, increase in MD and (5) an unconstraint model assuming no particular ordering of the group means. Bayes factors and PMPs of all five hypotheses are shown in Table 3.

**Table 3:** Bayes factors and posterior model probabilities on development of synaptic plasticity with age and interference of maternal deprivation.

Hypotheses		Bayes factor	PMP
Males			
(1) Increase in control and MD	μ_control P8-9_ < μ_control P22-24_ < μ_control P85-95_, μ_MD P8-9_ < μ_MD P22-24_ < μ_MD P85-95_	14.95	0.34
(2) Plateau in control and MD	μ_control P8-9_ < μ_control P22-24_ = μ_control P85-95_, μ_MD P8-9_ < μ_MD P22-24_ = μ_MD P85-95_	5.23	0.12
(3) Increase in control, plateau in MD	μ_control P8-9_ < μ_control P22-24_ < μ_control P85-95_, μ_MD P8-9_ < μ_MD P22-24_ = μ_MD P85-95_	18.32	0.42
(4) Plateau in control, increase in MD	μ_control P8-9_ < μ_control P22-24_ = μ_control P85-95_, μ_MD P8-9_ < μ_MD P22-24_ < μ_MD P85-95_	4.61	0.10
(5) Unconstraint	μ_control P8-9_, μ_control P22-24_, μ_control P85-95_, μ_MD P8-9_, μ_MD P22-24_, μ_MD P85-95_	-	0.02
Females			
(1) Increase in control and MD	μ_control P8-9_ < μ_control P22-24_ < μ_control P85-95_, μ_MD P8-9_ < μ_MD P22-24_ < μ_MD P85-95_	16.39	0.37
(2) Plateau in control and MD	μ_control P8-9_ < μ_control P22-24_ = μ_control P85-95_, μ_MD P8-9_ < μ_MD P22-24_ = μ_MD P85-95_	1.99	0.04
(3) Increase in control, plateau in MD	μ_control P8-9_ < μ_control P22-24_ < μ_control P85-95_, μ_MD P8-9_ < μ_MD P22-24_ = μ_MD P85-95_	1.45	0.03
(4) Plateau in control, increase in MD	μ_control P8-9_ < μ_control P22-24_ = μ_control P85-95_, μ_MD P8-9_ < μ_MD P22-24_ < μ_MD P85-95_	23.59	0.53
(5) Unconstraint	μ_control P8-9_, μ_control P22-24_, μ_control P85-95_, μ_MD P8-9_, μ_MD P22-24_, μ_MD P85-95_	-	0.02

Bayes factors were calculated by testing each hypothesis against the unconstraint model. The posterior model probabilities (PMPs) of all tested hypotheses add up to 1 and should be interpreted within the context of the total set of hypotheses.

Hypotheses 1–4 were all better supported by the data than the unconstraint model, indicating that there is indeed a particular ordering of the group means. Hypotheses 3 (increase in control, plateau in MD) and 1 (increase in control and MD) received the most support from the data (PMPs 0.42 and 0.34, respectively). To compare the support for one hypothesis over another, we divided the Bayes factors of the two models. More specifically, for hypothesis 3 (Bayes factor 18.32) compared to hypothesis 1 (Bayes factor 14.95), a ratio of 18.32/14.95 = 1.23 was obtained, i.e. hypothesis 3 is 1.23x better supported by the data than hypothesis 1. This suggests some preference for stating that in males MD accelerates maturation.

When performing the same Bayesian analysis with the female data, a clear contrast with the male data was seen in both the developmental effect in controls as well as the MD effect on development. With a PMP of 0.53, hypothesis 4 (plateau in control, increase in MD) was strongly favored over the other hypotheses. However, it should be noted that MD females did not reach adult levels of LTP slower compared to controls, but instead showed an overshoot in LTP magnitude at P85-95. This may suggest that both the development of synaptic plasticity as well as the response to MD is differently regulated in males and females.

## Discussion

In the current study we examined 1) the effect of MD on baseline fEPSP properties and synaptic plasticity at different stages of development, 2) whether these effects are sex-dependent, and 3) how these effects behave under high-stress conditions, mimicked *in vitro* by CORT incubation. Basal fEPSP size strongly increased with age, accompanied by a decrease in the required stimulation intensity; this was largely unaffected by MD and/or CORT treatment. Synaptic plasticity developed from LTD, at the start of the second postnatal week, towards LTP in young adult animals. There is some support for the notion that MD causes quicker maturation of synaptic plasticity in males, because LTP was significantly enhanced by MD at P22-24. Females showed an overshoot in LTP magnitude at adult age only.

### Deprivation Model

Maternal deprivation is a well-established and widely used model for early life stress in rats[38]. In our hands, MD induced alterations in hippocampal synaptic plasticity without accompanying long-term neuroendocrine effects. This is in accordance with earlier behavioral studies, where effects of MD were found at adult age while stress-sensitive parameters were mostly unaffected at that time[13,17]. The immediate reduction in body weight after MD was rapidly restored, which indicates that the animal is well able to cope with the initial metabolic challenge. In addition, the absence of large MD effects on stress-sensitive parameters allows us to compare the two treatment conditions without confounding effects of the state of the endocrine system.

### Developmental Increase in Synaptic Strength after HFS in Males and Females

In line with our expectations and earlier studies[39,40], extremely small baseline fEPSPs were observed at P8-9, accompanied by a high stimulation intensity. In general, it turned out to be difficult to obtain stable and reliable signals at this young age, which led us to combine data from male and female animals, after verifying that (at this age) there was no difference between the two sexes. Because of the comparability of the signals we have no indication that pooling of the datasets influenced our conclusions.

At this age, both glutamatergic and γ-Aminobutyric acid(GABA)-ergic transmission are still under development[40,41]. GABA switches towards the end of the second postnatal week from an excitatory state to being the main inhibitory neurotransmitter of the rodent brain[42]. Glutamate transmission, involving α-amino-3-hydroxy-5-methyl-4-isoxazolepropionic acid (AMPA) receptors, also undergoes developmental changes[39,43]. However, the underdeveloped structural morphology or CA1 pyramidal neurons at this age also likely contributes to the small responses. Dendritic length and complexity are low at birth and strongly increase during the early postnatal period[44]. Half of the synapses in the CA1 region occur on dendritic shafts instead of on spines during the first postnatal week[45]. In addition, the number of synapses greatly increases until the second postnatal week. All of these structural changes together gradually increase neuronal connectivity, contributing to the larger fEPSP amplitudes observed later in development.

At P8-9, we also observed LTD instead of LTP, as expected[29,30]. In addition to the shift in AMPA / N-Methyl-D-aspartate (NMDA) ratio[40], this is also due to a transient upregulation of AMPA receptor subunit GluA4 in CA1 pyramidal neurons when synaptic connectivity is forming; this subunit is involved in the switch from protein kinase A (PKA)- to Ca2+/calmodulin-dependent protein kinase II (CaMKII)-dependent LTP induction that occurs at the start of the second postnatal week[30,46,47]. Also, NMDA receptor subunits switch from GluN2A to primarily GluN2B around the same time[48]. Altogether, our data confirm the current views on the developmental state of signal transmission in the first postnatal week[49], both regarding basal transmission and synaptic plasticity.

### Sex-Dependent Effects of MD on Development

The developmental increase in synaptic plasticity after HFS was seen regardless of MD and in both sexes. However, in addition to this developmental effect, MD facilitated LTP induction in P22-24 males and in P85-95 females. It should be noted that all animals were weaned at P21, just before recordings on P22-24. Weaning is highly stressful for young rats, and we cannot exclude that the effect of MD in the male adolescent group was amplified by ‘weaning stress’ one to three days before recording. The higher basal CORT levels in P22-24 rats (when compared to adult animals) seem to support this; however, CORT is known to be higher in adolescence than in adulthood[50,51]. In future experiments, both weaned and non-weaned rats at P22-24 should be compared to judge the contribution of MD on LTP induction, in the absence of ‘weaning effects’.

Our findings on MD effects on LTP in the CA1 differ from results reported by Oomen et al.[13,14], who found an interaction between MD and CORT in adult males and no effect of MD or CORT in adult females. In males, the difference between the two studies can be explained by at least two factors. First, the study by Oomen et al.[13] was carried out in the dentate gyrus, whereas the present study was performed in the CA1 region. Earlier studies already demonstrated that the influence of early life environment on synaptic plasticity may differ between the dentate and CA1 area[52,53]. Second, the CORT treatment protocol was dissimilar: Oomen et al. washed in CORT 10 minutes before LTP induction, which coterminated with the applied theta burst stimulation. In contrast, we now incubated the slices with CORT for 20 minutes and waited at least 1h before applying high frequency stimulation. Consequently, Oomen et al. assessed the rapid nongenomic effects of CORT, presumably mediated by membrane-bound MRs and GRs, while we looked at slow genomic effects, for which a translocation of dimerized GRs to the nucleus is required (see [54] for review).

In our statistical models, the situation predicting an increase in LTP up to adulthood in controls and a plateau in the (male) MD groups yielded the highest Bayes factor, which we carefully interpret as support for an accelerated maturation after MD in males, at least regarding LTP. The basis for this model is a recent study by Bath et al., who also reported accelerated maturation after early life stress with regard to the expression of markers for cell proliferation in the hippocampus, NMDA receptor subunit expression and contextual fear conditioning[32]. In addition, several other early life stress models demonstrated accelerated development of microvessels in the hippocampus[55], differentiation and distribution of microglia throughout the brain[56] as well as the switch from PKA to CaMKII dependency of LTP[57]. However, replication experiments are required to confirm the presence of a potential MD-induced acceleration of maturity in hippocampal synaptic plasticity, given to the relatively small preference for this hypothesis over the one stating an increase of synaptic strength up into adulthood after MD in our experiment.

The pattern of MD interference with development of synaptic plasticity in females was different from that in males. Control females reached a plateau in synaptic strength already at P22-24, similar to the MD males. Acceleration of synaptic plasticity maturation by MD was not observed; in contrast, MD affected synaptic plasticity in adult females by further increasing the level of induced LTP.

Sex-dependent developmental effects of MD in the hippocampus were earlier seen when focusing on neurogenesis[19]: MD males showed an increase in neurogenesis at P21 and a decrease at 10 weeks of age compared to controls[13,58], while females showed a decrease at P21 and no effect of MD at 10 weeks[14,58]. Adult synaptic plasticity in the same region was earlier shown to be facilitated in adult males[13], with no effect in adult females[14]. Together, these earlier and the current studies show that the effects of MD are strongly dependent on the age at which the effect is assessed as well as on the sex of the animal. This emphasizes the importance of determining effects of early life adversity at different ages and in both sexes to get a complete view of the time course of effects.

### CORT Effects on Synaptic Plasticity

In contrast to earlier studies[13] there was no interaction between CORT and MD treatment, although there was a trend in P85-95 females. An interaction would have been in line with the match-mismatch hypothesis, which states that individuals perform optimally when the later-life environment matches early life experience regarding stress levels[22]. It should be noted that earlier studies on the match-mismatch theory in rats generally focused on older ages (>3 months of age), whereas we studied juvenile and young adult rats only. We cannot exclude that the interaction between CORT and MD is more outspoken at later stages in life.

Regardless of MD, we also performed an exploratory analysis of the effect of CORT in control animals, to allow comparison with earlier studies (for review see Kim et al.[59] and Joëls et al.[54]). We observed a CORT-induced suppression of LTP induction in adult males, which is in line with many previous studies[34–36], lending support to the quality of the current dataset. In none of the other groups did CORT change LTP induction, although a suppression of LTP at trend level was observed in females at P85-95. This is in line with data from Qiao et al. who did find a suppression of LTP by CORT in the CA1 hippocampus of female rats[60]. It should be noted that in the latter study slices were continuously perfused with CORT in aCSF during the recording, thereby combining fast and slow CORT effects.

Plasma CORT from trunk blood and induced %LTP were correlated in the P85-95 male rats, where we observed (in agreement with the group averages) a negative correlation. Of note, the suppressive effect by CORT on LTP in adult males was observed with very high CORT levels, an order of magnitude above the levels seen in our control (MD and no-MD) rats, because the latter were always killed under rest and in the morning when plasma CORT levels are low. Spontaneous fluctuations in basal CORT levels may therefore not strongly correlate with the ability to induce LTP. This may explain why the result of correlational analyses in the other experimental groups, e.g. P22-24 males, did not fit with the observations on group averages. Alternatively, plasma CORT levels may not be the only or main driving factor behind LTP facilitation by MD. Possibly, structural rearrangements of dendrites (as seen in the DG[13]), increased spine densities and/or an increase in the number of AMPA receptors available to incorporate into the synapse after HFS could better explain the observed effects. It would be interesting to determine in future studies whether these changes indeed take place after MD.

The somewhat weaker effect in females that we observed could be due to circulating sex steroids, modulating the effect of CORT on LTP induction. However, there was no effect of estrous cycle stage on LTP induction. This is in line with a study from Warren et al.[61], who showed a doubled LTP magnitude in the pro-estrus stage compared to other stages only when rats had been decapitated in the afternoon. Since all our animals were decapitated in the morning, the lack of estrus cycle effects is not without precedent.

## Conclusion

In conclusion, our study indicates that MD affects the development of synaptic plasticity in the CA1 hippocampus in a sex-dependent manner. This implies that the optimal timing for treatments against the detrimental effects of early life stress is likely to be different in males and females. A better understanding of the time course of hippocampal development after stressful early life experiences and its interplay with sex or gender is required to develop targeted therapies.
